# Time to Diabetic Retinopathy and Its Risk Factors among Diabetes Mellitus Patients in Jimma University Medical Center, Jimma, Southwest Ethiopia

**DOI:** 10.4314/ejhs.v32i5.9

**Published:** 2022-09

**Authors:** Gurmessa Nugussu Gelcho, Firomsa Shewa Gari

**Affiliations:** 1 Department of Statistics, College of Natural Science, Jimma University, Jimma, Oromia, Ethiopia; 2 Department of Statistics, College of Natural and Computational Science, Assosa University, Assosa, Benishangul Gumuz, Ethiopia

**Keywords:** Diabetic retinopathy, Retrospective study, Cox proportional hazard model, Median time

## Abstract

**Background:**

Diabetic retinopathy is a complication of diabetes, caused by high blood sugar levels damaging the eye. Globally, diabetic retinopathy affects more than 103.12 million people. Diabetic retinopathy is among the leading causes of vision loss at the global level, including in Ethiopia. Therefore, the study aimed to assess the time to develop diabetic retinopathy and identify factors associated with diabetic retinopathy among diabetes patients.

**Methods:**

A retrospective study was conducted from September 1, 2021 to January 30, 2022. Data was collected using semistructured questionnaire. The Cox proportional hazard model were used to determine the median time to develop diabetic retinopathy and identify predictors of diabetic retinopathy. Data was analyzed using R software.

**Results:**

A total of 373 diabetes patients were included in this study. The prevalence of diabetic retinopathy was 41.3%. The median time was 41 months, ranging from 39 to 73 months. Elder age (HR=3.17, 95%CI: 1.53, 6.58), being male (HR=2.34, 95%CI: 1.35, 6.15), previous family history of diabetes (HR=4.16, 95%CI: 2.19, 8.37), longer duration of diabetes (HR=2.86, 95%CI: 1.41, 5.31) received only insulin therapy (HR=3.91, 95%CI: 1.36, 7.94), and high systolic blood pressure(HR=2.32; 95%CI: 1.12, 4.39) were statistically significant factors related to development of diabetes retinopathy.

**Conclusions:**

More than half of diabetic patinets in this study were developed retinopathy diabetes within a few months of being diagnosed. As a result, we advocate that the best way to preserve our vision from diabetic retinopathy is to maintain our diabetes under control, and the high-risk population receive early screening for diabetes.

## Introduction

Diabetes Retinopathy (DR) is a complication of diabetes that affects the retina of the eye due to excessive blood sugar levels (retina). It is a collection of chronic metabolic diseases characterized by a failure to produce insulin or insulin resistance, or both (1–3). Diabetic retinopathy is the most common micro vascular problem in people with diabetes, and it can cause irreparable vision loss. No proliferative diabetic retinopathy (NPDR) and proliferative diabetic retinopathy (PDR) are the two types of diabetic retinopathy that affect the retina (PDR). About 95 million (35.4 percent) diabetes patients worldwide have DR, with a third having vision-threatening DR and 7.6% having retinal edema. The global yearly incidence of DR ranges from 2.2 percent to 12.7%, with a progression rate of 3.4 percent to 12.3 %(4–5).

According to the International Diabetes Federation, the number of diabetic adults in Africa would rise from 19.4 million in 2019 to 47.1 million in 2045 (6). Globally, the prevalence of DR in diabetes mellitus (DM) patients is estimated to be 27.0% (7). The prevalence of DR is reported to be 31.6 percent in Africa (8) and 19.48 percent in Ethiopia (9) based on a pooled analysis of several hospital-based studies. According to the World Health Organization (WHO), DR is responsible for 37 million cases of blindness worldwide (10).

Diabetic retinopathy is the leading cause of new incidences of blindness in those aged 20 to 74. Nearly all patients with type1 diabetes and more than 60% of patients with type2 diabetes develop retinopathy throughout the first two decades of their condition. Diabetic retinopathy was responsible for 86 percent of blindness in the younger-onset group (11). Diabetic retinopathy was responsible for one-third of the instances of legal blindness in the older-onset group, where other eye illnesses were common. Blindness affects 1.5 billion people worldwide, with 0.4 million people suffering from DR. DR-related blindness has increased from 0.2 million to 0.4 million, with moderate-to-severe visual impairment increasing from 1.4 million to 2.6 million from 1990 to 2015 (12).

According to different studies, the most consistent factors associated with the development of DR in diabetic patients were longer duration of diabetes (13–17), gender (13,18), high fasting blood sugar level (19–20), presence of hypertension (13,21), systolic blood pressure (1415,17), glycine hemoglobin (16–17), being on insulin treatment alone (22–24), family history of diabetes (25–26), and poor socioeconomic status (27–28).

Consistently elevated blood glucose levels produce broad vascular damage, which leads to a variety of macro and micro vascular problems. Diabetic Retinopathy is a micro vascular consequence of diabetes that affects the eyes over time. Diabetic Retinopathy progresses from its milder abnormalities to its advanced stages if it is not detected and treated early. Macular edema, traction retinal detachment, and nonvascular glaucoma worsen diabetic retinopathy, resulting in substantial visual impairment.

However, there is little researches on prevalence and its risk factor of diabetic retinopathy in Ethiopia, specially in Jimma. Furthermore, no previous study in the study area using survival data with an appropriate survival model. While using classical regression to analyze survival data, the outcome is biased. As a result, the goal of this study was to determine the median time to develop retinopathy, the prevalence and associated factors of DR in diabetic patients at Jimma University Medical Center.

## Methods

**Study area and period**: The research was conducted at Jimma University Medical Center (JUMC) in Jimma Town, Southwest Ethiopia. The hospital's diabetes clinic is one of numerous chronic follow-up clinics that take place twice weekly. Internists, medical residents, medical interns, and general nurses provide the service. The study was carried from September 1, 2021 to January 30, 2022 at JUMC.

**Study design**: A retrospective cohort study design was used.

**Study population**: All patients newly diagnosed with DM at JUMC during follow-up visits between September 1, 2021 to January 30, 2022.

**Inclusion and exclusion criteria**: All patients who had at least one follow up visit and adults (aged ≥ 15 years) were included. Patients who lose follow up, who developed diabetic neuropathy, below 15 years age, and died before the end of this study period were excluded.

**Sample size determination**: Sample size was calculated by using a single population proportion formula by taking 41.4% proportion of DR from previous research done in Jimma University Medical Center, Southwestern Ethiopia (29). Accordingly, a sample size of 373 was obtained. The sample units were chosen using systematic random sampling.

**Diagnosis of diabetic retinopathy**: Diabetic retinopathy diagnosis includes a medical history, an ophthalmic examination and screening with retinal photographs, and regular follow up. In short, diabetes retinopathy was defined by both direct and indirect ophthalmoscopy assessments done by retinal specialists confirmed by fundus photography. Diabetes retinopathy occurs when blood vessels in the retina and optic nerve are damaged using dilated eye exam (drops are placed in the eyes to widen or dilate the pupils) using a special magnifying lens. Diabetes retinopathy apparatus if there is leaking blood in retinal vessels, Pale, fatty deposits on the retina indicating leaking blood vessels. The incidence of DR was determined from the start of diagnosis date of DM until the last follow-up visit or known date of DR occurrence (18).

**Variables in the study**: The outcome variable was time to DR due to DM after dilated eye examination using tonometry and ophthalmologic instruments. The independent variables for this study were gender, age, education level, family wealth index, family monthly income, residence, smoking status, religion, occupation, drinking alcohol status, diabetes duration, hypertension, family history of DN, history of eye examination, glycemic HbAl1c, types of DM, mode of treatment, and systolic blood pressure.

**Data collection method**: A well-structured questionnaire was developed and distributed. The questionnaire was developed in English and then translated into Afan Oromo before being returned to English to confirm its reliability and uniformity.

**Data quality assurance**: To ensure that the checklist was adequate, an initial evaluation was conducted prior to the actual data collection period. As a result, the checklist's variables that were unavailable were eliminated. Data was collected by skilled health-care providers who had experience with chronic illness earlier. The data quality was double-checked at many points throughout the investigation. The data was then inputted, exported, analyzed, and interpreted after the questionnaires were validated, coded, and processed.

**Statistical methods and data analysis**: The data was cleaned, coded, and entered into EPI info (version 7.0) before being exported to R software (version 4.1.3) for analysis. The Cox proportional hazard model is used for multivariate analysis of time to develop diabetic retinopathy and to identify factors associated with diabetic retinopathy among diabetes mellitus patients at JUMC. The Cox proportional hazards model given by *λ*(*t*|*Z*) = *λ*_0_(*t*)*e^ZTβ^*, where, *Z* = (*Z*_1_, *Z*_2_, .., *Z_p_*)^*T*^ and *β* = (*β*_1_, *β*_2_, ..., *β_p_*)^*T*^ Zis px1vector of covariates such as treatment indicators and prognostic factors, and *β* is a × 1 vector of regression coefficients. Finally, a hazard ratio with a 95 percent confidence interval was calculated, and factors with a p-value less than 0.05 were consideredstatistically significant factors of diabetic retinopathy.

**Operational definitions**

**Diabetic neuropathy**: Diabetic retinopathy is caused by damage to the blood vessels in the tissue at the back of the eye (retina).

**Time to diabetic retinopathy**: The time it takes from being diagnosed with diabetes to getting diabetic retinopathy in a month from the patient's card.

**Censored**: Patients who did not acquire diabetic retinopathy throughout the study period, or who died, were lost to follow-up, or were transferred out before developing diabetic retinopathy were excluded from the study were considered as censored patients.

**The event of interest**: The experience of symptoms of diabetic retinopathy within the follow-up period.

**Ethics approval and consent to participate**: Ethical approval was obtained from the Institutional Research Ethics Review Committee of the Jimma University College of Natural Sciences. A letter of support was written for the JUMC. The authors submitted an official letter to JUMC. After clarifying the purposes of the study, informed written permission was obtained from all DM, and namelessness and privacy of the data were kept. Respondents have the right not to participate or withdraw from the study at any stage.

## Results

**Socio-demographic and economic characteristics**: Three hundred seventy three DR patients were included in the study. The median age of the diabetic patients at the time of diabetic diagnosis was 41 months with a range of 39 to 73 months. From the 373 study patients, 138 (37.0%) were in the age category of 48 and higher. Two hundred two (54.1%) and 250 (67.0%) were male and rural residents, respectively. Among the total patients in the study, 293 (78.5%) and 135(36.2%) were married and had primary education, respectively. The study findings showed that majority of the patients were non-government employees (51.8%). Nearly half of the participants in the study, 165 (44.2 percent), were Muslim (Table 1).

**Table 1:** Socio-demographic and economic characteristics of the study participants at JUMC (n=373)

Variables	Category	Event(DR) n (%)	Censore dn (%)	Total n (%)	Median (in months) (95%CI)
Gender	Male	90(24.1)	112(30.0)	202(54.1)	75(72,79)
	Female	64(17.2)	107(28.7)	171(45.9)	76(73,80)
Age	≤27	25(6.7)	51(13.7)	76(20.4)	69(64,77)
	28–37	19(5.1)	47(12.6)	66(17.7)	71(68,81)
	38–47	39(10.5)	54(14.5)	93(25.0)	76(70,88)
	≥48	71(19.0)	67(18.0)	138(37.0)	81(72,91)
Residence	Rural	97(26.0)	153(41.0)	250(67.0)	79(74,85)
	Urban	57(15.3)	66(17.7)	123(33.0)	71(68,79)
Marital status	Single	29(7.8)	51(13.7)	80(21.5)	63(60,69)
	Married	125(33.5)	168(45.0)	293(78.5)	67(60,76)
Education level	No education	64(17.2)	82(22.0)	146(39.2)	74(71,82)
	Primary school	59(15.8)	76(20.4)	135(36.2)	74(70,81)
	Secondary and above	31(8.3)	61(16.4)	92(24.7)	71(68,79)
Occupation status	Gov't employee	46(12.3)	59(15.8)	105(28.1)	68(66,76)
	Non-gov't employee	79(21.2)	114(30.6)	193(51.8)	75(70,79)
	No job	30(8.0)	46(12.3)	76(20.3)	70(67,79)
Religion	Orthodox	37(9.9)	61(16.4)	98(26.3)	81(76,85)
	Muslim	63(16.9)	102(27.3)	165(44.2)	78(74,82)
	Protestant	24(6.4)	32(8.6)	56(15.0)	76(72,85)
	Others	30(8.0)	24(6.4)	54(14.4)	78(76,83)
Family monthly income	≤2000	71(19.0)	78(20.9)	149(39.9)	68(66,73)
(Ethiopian Birr)	2001–3577	30(8.0)	61(16.4)	91(24.4)	73(71,77)
	3578–6500	34(9.1)	43(11.5)	77(20.6)	68(66,72)
	≥6501	19(5.1)	37(9.9)	56(15.0)	71(68,76)

**Behavioral, clinical, and diabetic related characteristics**: Regarding prevalence of alcohol intake, 130(34.8) consumed alcohol. Two hundred and twenty-eight (34.3%) and 113(30.2%) had history of hypertension and family history of diabetes mellitus, respectively. Only one hundred and nine (29.2%) patients had history of eye examination. Nearly half of the patients had normal body mass index (45.5%). Majority of the patients, (219(58.7%) had DM more than five years. 210(56.3%) and 159(42.6%) had type1 DM and took only insulin for treatment, respectively (Table 2).

**Table 2:** Behavioral, and clinical related characteristics of the study participants at JUMC (n = 373)

Variables	Category	Event(DR) n (%)	Censored n (%)	Total n (%)	Median (in months)(95%CI)
Drinking alcohol	Yes	53(14.2)	77(20.6)	130(34.8)	78(73,85)
	No	101(27.1)	142(38.1)	243(65.2)	76(71,78)
Smoking status	Yes	46(12.3)	60(16.1)	106(28.4)	80(76,83)
	No	108(28.9)	159(42.6)	267(71.6)	77(75,80)
History of	Yes	48(12.9)	80(21.4)	128(34.3)	82(80,85)
hypertension	No	106(28.4)	139(37.3)	245(65.7)	72(70,76)
Family history of DM	Yes	39(10.4)	74(19.8)	113(30.2)	83(80,86)
	No	115(30.8)	145(38.9)	260(69.8)	73(70,78)
History of eye exam	Yes	43(11.5)	66(17.7)	109(29.2)	78(75,83)
	No	111(29.7)	153(41.0)	264(70.8)	76(74,78)
Glycemic (HbAl1c)	≤7%	58(15.5)	51(13.8)	109(29.3)	68(65,75)
	>7%	96(25.7)	168(45.0)	264(70.7)	73(69,78)
Body mass index	≥24.9	22(5.9)	73(19.6)	95(25.5)	64(60,67)
	25–29.9	71(19.0)	99(26.5)	170(45.5)	71(65,74)
	≥30	61(16.3)	47(12.7)	108(29.0)	76(74,79)
Duration of DM in	<5	94(25.2)	60(16.1)	154(41.3)	81(78,84)
years	>=5	60(16.1)	159(42.6)	219(58.7)	83(80,87)
Types of DM	Type I	81(21.7)	129(34.6)	210(56.3)	61(59,67)
	Type II	73(19.6)	90(24.1)	163(43.7)	76(69,80)
Mode of treatment	Insulin alone	75(20.1)	84(22.5)	159(42.6)	66(63,71)
	Non-insulin	32(8.6)	67(18.0)	99(26.6)	69(67,73)
	Mixed	47(12.6)	68(18.2)	115(30.8)	68(66,73)
Systolic blood	<140	81(21.7)	115(30.8)	196(52.5)	67(64,71)
pressure	≥140	73(19.6)	104(27.9)	177(47.5)	71(65,74)

**Prevalence and factors associated with diabetic retinopathy**: Among 373 patients, 154(41.3%) had diabetic retinopathy. In multivariate survival analysis, all statistically significant factors with a p-value below 0.25 in univariate survival model were included. Age, residence, previous history of hypertension, family history of DM, duration of DM, type of treatment used, and systolic blood pressure were all related risk factors of diabetic retinopathy (p-value<0.05).

From multivariate survival analysis using Cox proportional hazard model, the estimated hazard ratio of DR was 3.17 times higher in age category 40 and higher years old DM patients than less than 27 years while holding other covariates constant (HR=3.17,95% CI: 1.53,6.58). Males had a 2.34 fold increased risk of DR when compared to females (HR=2.34, 95%CI: 1.35, 6.15). The risk of acquiring DR was 4.16 times higher in patients whose families had a history of diabetes than in patients whose families had no history of diabetes (HR=4.16, 95%CI: 2.19, 8.37).

Keeping other variable constant, the hazard of diabetic retinopathy was increased by 2.86 times higher among patients who had DM 5 years and above as compared to less than 5 years (HR=2.86, 95%CI: 1.41, 5.31). Patients who received only insulin therapy had a 3.91-fold higher risk of developing diabetes than those who received both insulin and tablet medication (HR=3.91, 95%CI: 1.36, 7.94). Holding all other variables constant, the risk of DR was 2.32 times higher in patients with systolic blood pressure greater than 140 mmHg compared in those with systolic blood pressure less than 140 mmHg (HR=2.32; 95%CI: 1.12, 4.39) (Table 3).

**Table 3:** Multivariate analysis of characteristics of study participants at JUMC, 2022 (n = 373)

Variables	Event(DR) n (%)	Censored n (%)	HR (95%CI)	p-value
**Age of patients**				
≤27 (Ref)	25(6.7)	51(13.7)	1	
28–37	19(5.1)	47(12.6)	2.25(0.71,4.87)	0.128
38–47	39(10.5)	54(14.5)	1.39 (0.54,3.76)	0.071
≥48	71(19.0)	67(18.0)	3.17(1.53,6.58)	0.026*
**Gender**				
Male	97(26.0)	153(41.0)	2.34(1.35, 6.15)	0.034*
Female (Ref)	57(15.3)	66(17.7)	1	
**Family history of DM**				
Yes	39(10.4)	74(19.8)	4.16 (2.19,8.37)	0.004*
No(Ref)	115(30.8)	145(38.9)	1	
**Duration of DM**				
<5 years (Ref)	94(25.2)	60(16.1)	1	
≥5 years	60(16.1)	159(42.6)	2.86(1.41, 5.31)	0.007*
**Mode of treatment**				
Insulin alone	75(20.1)	84(22.5)	3.91(1.36,7.94)	0.016*
Non-insulin	32(8.6)	67(18.0)	1.41(0.78,4.86)	0.147
Mixed (Ref)	47(12.6)	68(18.2)	1	
**Systolic blood pressure**				
<140 mmHg (Ref)	73(19.6)	104(27.9)	1	
≥140 mmHg	81(21.7)	115(30.8)	2.32 (1.12,4.39)	0.031*

* Significant at 5%; SE: Standard error; HR: Hazard ratio; Ref: Reference group

## Discussion

Mild non-proliferative retinopathy (the first stage of diabetic retinopathy) is the most common early stage of diabetic retinopathy, which may not require treatment, but we must maintain our diabetes under control. If the diagnosis is delayed at this stage, it progresses to the second stage (moderate non-proliferative retinopathy) and then to the advanced stage (proliferative diabetic retinopathy). This study endeavored to estimate time to develop diabetic retinopathy, determine the prevalence of diabetes retinopathy and identify the factors associated with DR among DM patients at Jimma University Medical Center, Jimma, Ethiopia. The prevalence of DR was 41.3 percent, and the median duration for DM patients to develop DR was 41 months, ranging from 39 to 73 months. This study found a higher frequency of DR than those conducted in Northwest Ethiopia, Egypt, Dakar, and China (18, 25, 30–31). This discrepancy could be attributable to differences in health-care systems, sample sizes, patient duration of diabetes, and diabetes-care quality. However, in this study, the prevalence rate was lower than in a study conducted at Tikur Anbessa Hospital in Ethiopia, Zambia, India, and Sudan (18, 33–35). This mismatch could perhaps be explained by the study's data gathering time, and sample size.

This study found that among DM patient's males had a shorter time than females to develop diabetes retinopathy. This finding are in agreement with research carried in Tikur Anbessa, Ethiopia, and China (18, 36). This could be because adult males have more defective neuroretinal function than adult females (37). However, the study conducted in Egypt found that males had a lower risk of developing micro vascular complications of diabetes than females (30).

According to this study, diabetic patients with a family history of diabetes have a higher risk of developing DR. This is similar to studies done in Arbaminch, Southwest Ethiopia, and China, which found that diabetic patients with a family history of diabetes were more likely to develop micro vascular complications such as DR (25, 38–39).

According to the findings of this study, longer duration of diabetes increases the likelihood of developing DR. This finding is consistent with studies in Tikur Anbessa, Arbaminch, Jimma, Egypt, Dakar, and China (18–19, 25, 30–33), which found that having DM for a longer period of time is significantly associated with the development of DR. This association could be explained by the fact that widening of the retinal artery occurs as the duration of diabetes increases, which is a subclinical marker of endothelial dysfunction that leads to DR.

Moreover, the presence of DR was higher in diabetic patients who received only insulin therapy versus those who received both insulin and tablet. This was consistent with the findings of studies done in Tikur Anbessa, Los Angeles, Brazil, and Northwest Ethiopia (18, 15–16, 40), which found that diabetic patients receiving insulin therapy alone had an increased risk of developing DR. It has been suggested that combined therapy can achieve a better and faster achievement of a good glycemic target, which aids in the prevention of long-term diabetes complications such as DR. In this study diabetic patients with Systolic Blood Pressure greater than 140 mmHg were also one important factor in the development of DR among diabetic patients. Comparable studies have been conducted in Australia, China, Arbaminch, and Southwest Ethiopia (14, 17, 25, 38).

In conclusion, the prevalence of DR in a diabetic patients attending at JUMC was higher compared to the global and national prevalence DR. The most important risk variables related with the development of DR in diabetes were found to be age, gender, previous family history of DM, longer duration of diabetes, being on Insulin treatment alone, and higher systolic blood pressure. More than half of diabetic cases developed diabetic retinopathy within a few months of being diagnosed. As a result, we advocate that the best way to preserve our vision from diabetic retinopathy is to maintain our diabetes under control, and that high-risk populations receive early screening for diabetes and its complications.

As a result of the study's focus on hospital patients, it does not accurately represent the prevalence of DR in the general diabetic population. Because the study was retrospective, we missed some important diabetes complications that had a significant association with DR, such as chronic kidney disease, because we obtained the data through chart review.
